# Human Infections with *Pseudoterranova cattani* Nematodes, Chile

**DOI:** 10.3201/eid2110.141848

**Published:** 2015-10

**Authors:** Thomas Weitzel, Hiromu Sugiyama, Hiroshi Yamasaki, Cristian Ramirez, Reinaldo Rosas, Rubén Mercado

**Affiliations:** Clínica Alemana-Universidad del Desarrollo, Santiago, Chile (T. Weitzel, R. Rosas);; National Institute of Infectious Diseases, Tokyo, Japan (H. Sugiyama, H. Yamasaki);; Universidad Mayor, Santiago (C. Ramirez); Universidad de Chile, Santiago (R. Mercado)

**Keywords:** Parasitic diseases, parasites, human infections, Pseudoterranovosis, Anisakid nematode, Pseudoterranova cattani, Chile, zoonoses, foodborne illness, fish

**To the Editor:** Anisakidosis is an emerging foodborne zoonosis caused by nematode larvae of the Anisakinae subfamily, which includes the genera *Anisakis*, *Pseudoterranova*, and *Contracecum* (*1*). In natural cycles, anisakid larvae are transmitted to marine mammals or piscivorous birds when they eat raw saltwater fish or squid. In the human incidental host, larvae attach to the mucosa of the gastrointestinal tract, causing clinical features ranging from asymptomatic carriage to severe abdominal pain with complications, such as gastric perforation (*2*). Microscopical diagnosis is hampered by the lack of distinguishing morphologic characteristics in larval stages (*1*). Recently, molecular genetic techniques have shown that the main species, *Anisakis simplex* and *Pseudoterranova decipiens*, are in fact species groups with distinct geographic and biologic characteristics (*3*,*4*). The *P. decipiens* complex consists of at least 6 sibling species (Technical Appendix Table). We report 4 human infections with *P. cattani* diagnosed during 2012–2014. 

The case-patients were adults 22–59 years of age; 2 were female, and all lived in Santiago, Chile. Additional anamnestic and clinical data were available for 3 patients: all spontaneously regurgitated the parasites without having other gastrointestinal complaints. All 3 reported eating ceviche, a dish made of raw marine fish marinated in lemon juice. One patient reported a tingling sensation and coughs before the expulsion of a highly motile larva (Video). This patient was awaiting oral surgery after a bicycle accident and had eaten the last raw fish dish 2 weeks previously. Initially, parasites were identified by morphologic criteria. Larvae were 20 mm long, were of whitish to reddish color, and had 3 anterior lips (Technical Appendix Figure 1). Because of the presence of an anteriorly directed cecum (Technical Appendix Figure 2), they were assigned to *Pseudoterranova* species.

**Video:**
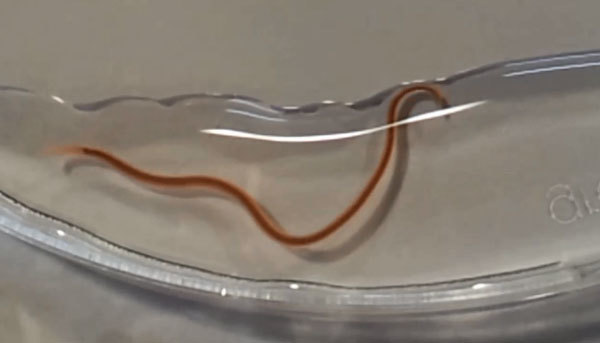
Actively mobile larva of *Pseudoterranova cattani* after oral expulsion.

For further molecular identification, DNA samples were extracted by using a DNeasy Blood and Tissue Kit (QIAGEN K.K., Tokyo, Japan). The rRNA gene containing 2 internal transcribed spacer (ITS) regions was amplified by PCR using primers NC5 and NC2, as previously described (*5*). PCR products were sequenced by using a BigDye Terminator Cycle Sequencing Kit (Applied Biosystems Inc., Foster City, CA, USA) on an automated sequencer (ABI3100, Applied Biosystems). Sequence similarities were determined by a BLAST search of DDBJ (http://blast.ddbj.nig.ac.jp/top-j.html). The GENETYX-WIN program version 7.0 (Software Development Co., Tokyo, Japan) facilitated sequence alignment and comparison. Within the 4 ITS sequences of amplicons obtained, all were 100% identical, and alignment with the other *P. cattani* sequence differed only in 1 nt. ITS sequences of 2 isolates are available in GenBank (accession nos. KF781284 and KF781285). All *P. cattani* sequences showed a previously described deletion of ≈14 bases (Table), which is not observed in other members of the *P. decipiens* species complex (*5*).

**Table:** Alignment (comparison) of nucleotide sequences of the ITS1 gene of *Pseudoterranova cattani* and the Chilean specimen and P. decipiens*

Isolate	ITS1 sequence at 240–270 nt	GenBank accession no.
Pc1	CTCTGTT--------------AACGCAGAGT	AJ413981
CL#3	CTCTGTT--------------AACGCAGAGT	KF781284
PdCa1	CTCTGTTTTGGTTTCAACGCTAACGCAGAGT	AJ413979

*CL#3, specimen from Chile; Pc1; ITS, internal transcribed spacer. Pc1, *P. cattani*; PdCa1, *P. decipiens*.

This study identified *P. cattani* as a parasite capable of infecting humans. The definitive natural host of this parasite is the South American sea lion, *Otaria byronia*. At least 4 species of coastal fish were described as intermediate or paratenic hosts, including popular Chilean food fish species, such as *Merluccius gayi*, *Genypterus maculatus*, and *Cilus gilberti* (*6*). The spectrum of species causing human pseudoterranovosis is uncertain because most cases were reported as *P. decipiens* (sensu lato) or *Pseudoterranova* sp. Only recently, 1 case of *P. azarasi* infection has been documented in a patient from Japan (*7*). Although comparative studies are lacking, *Pseudoterranova* larvae seem to be less invasive and cause milder symptoms than *Anisakis* larvae (*2*,*8*). In the cases reported here, larvae were spontaneously expelled without further symptoms, except in 1 patient who reported the typical feature of noninvasive pseudoterranovosis, also described as “tingling throat syndrome” (*8*), a foreign body sensation accompanied by cough and retching. In Chile, ≈30 human cases have been reported, all diagnosed as *P. decipiens* or *Pseudoterranova* sp. by morphologic criteria (*9*,*10*). Most patients described mild oropharyngeal complaints and cough. More severe manifestations similar to parasitic pharyngitis caused by *Fasciola hepatica* or *Linguatula serrata* seem to be absent, although 1 patient had symptoms of asphyxia (*9*). The extent to which these cases in Chile were caused by *P. cattani* is uncertain because molecular diagnosis was not performed. The length of stay and location within the human gastrointestinal tract of *Pseudoterranova* larvae are unknown, but as indicated by 1 case in our report, lack of symptoms for up to 2 weeks is possible.

These cases demonstrate that *P. cattani* is an incidental human parasite causing oropharygeal pseudoterranovosis. To better understand the epidemiology and clinical relevance of these emerging fishborne zoonotic infections, molecular diagnostic techniques need to be more widely applied, especially in regions where raw fish is part of the regular diet, such as in many parts of South America.

Technical Appendix Sibling species within the *Pseudoterranova decipiens* species complex and their geographic distribution; *P. cattani* larva with 3 anterior lips; anterior part of *P. cattani* larva showing anteriorly directed cecum.
