# Supplementary material for: Human Infections with Pseudoterranova cattani Nematodes, Chile
Source: Emerg Infect Dis. 2015 Oct;21(10):1874–5. doi: 10.3201/eid2110.141848 (PMC4593429; doi:10.3201/eid2110.141848)
Supplement: Technical Appendix — Sibling species within the Pseudoterranova decipiens species complex and their geographic distribution; P. cattani larva with 3 anterior lips; anterior part of P. cattani larva showing anteriorly directed cecum. [file 14-1848-Techapp-s1.pdf]

Article DOI: http://dx.doi.org/10.3201/eid2110.141848

Human Infections with *Pseudoterranova cattani* Nematodes, Chile

Technical Appendix

Technical Appendix Table. Sibling species within the *Pseudoterranova decipiens* species complex and their geographic distribution

Species	Geographic distribution
P. decipiens (sensu stricto)	North Atlantic, Northeast Pacific
P. bulbosa	Northeast Atlantic, Northwest Pacific
P. azarasi	North Pacific
P. krabbei	Northeast Atlantic
P. cattani	Southeast Pacific
P. decipiens E	Antarctic and sub-Antarctic waters

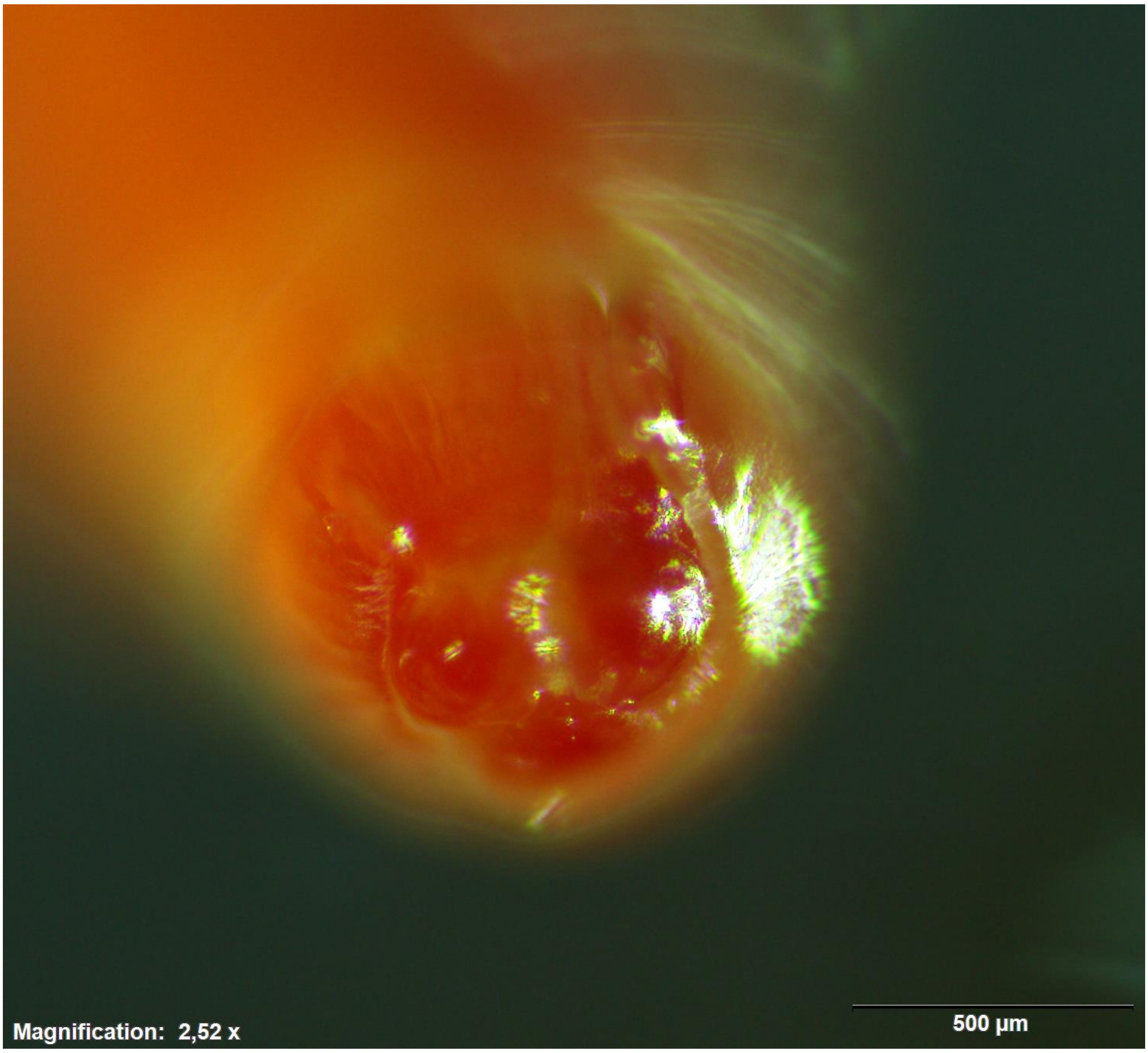

Technical Appendix Figure 1. *Pseudoterranova cattani* larva with 3 anterior lips.

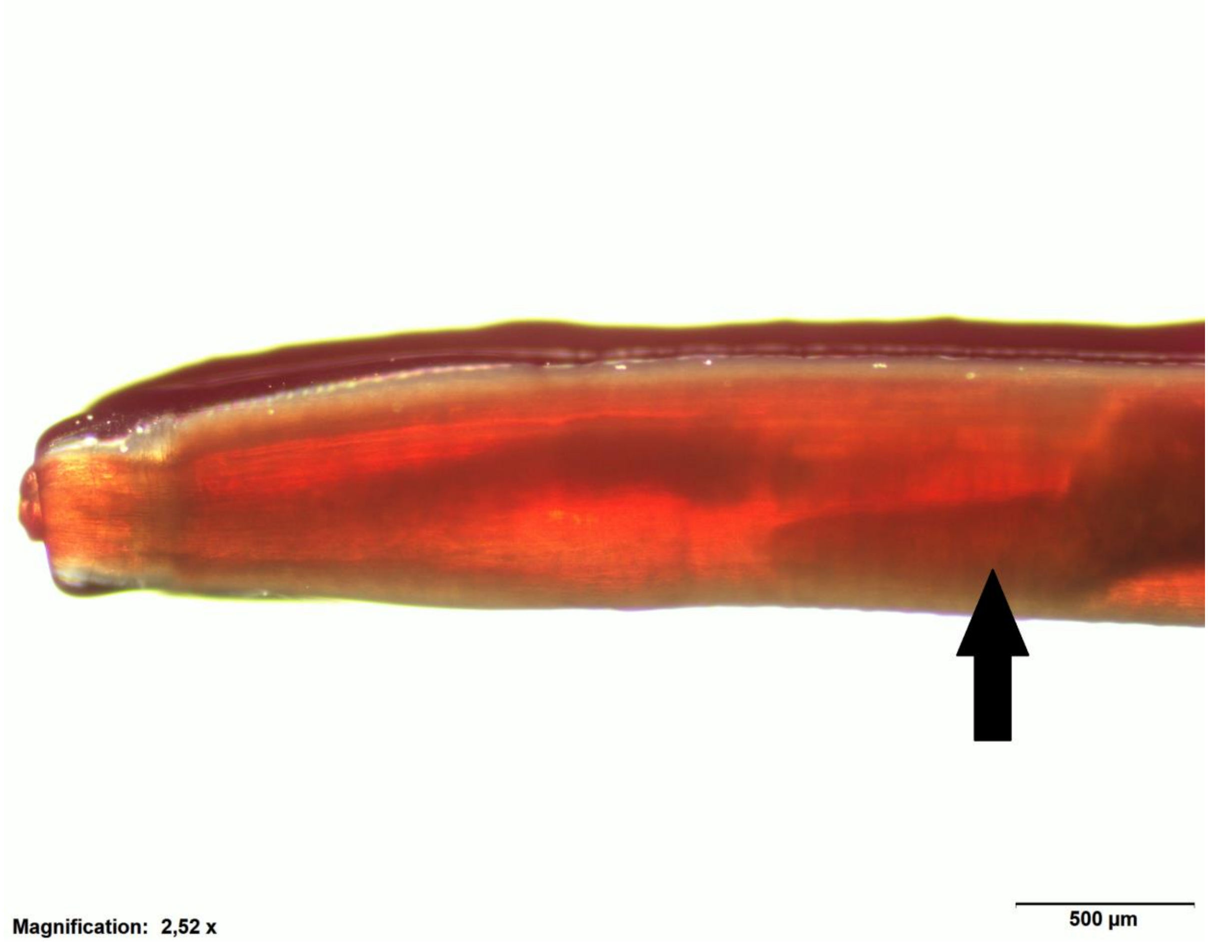

Technical Appendix Figure 2. Anterior part of *Pseudoterranova cattani* larva showing anteriorly directed cecum (arrow).
